# Supplementary material for: Molecular mechanisms of lipid metabolism abnormalities driving sepsis and atrial fibrillation: A Systematic study based on bioinformatics and machine learning
Source: PLoS One. 2025 Dec 8;20(12):e0338511. doi: 10.1371/journal.pone.0338511 (PMC12685191; doi:10.1371/journal.pone.0338511)
Supplement: S1 Table — (DOCX) [file pone.0338511.s002.docx]

Table S1 The expression profiles of common key genes across the two diseases

|  | id | logFC | AveExpr | t | P.Value | adj.P.Val | B |
| --- | --- | --- | --- | --- | --- | --- | --- |
| SEPSIS | CD81 | -1.267902986 | 6.196256438 | -14.71373004 | 2.50E-44 | 4.67E-43 | 89.83369549 |
|  | CKAP4 | 1.554647612 | 9.260536164 | 19.84940209 | 1.07E-73 | 2.50E-71 | 157.0615869 |
|  | DPEP2 | -1.262954301 | 7.546636785 | -9.761522207 | 1.60E-21 | 7.70E-21 | 37.77797 |
| AF | CD81 | -0.215798075 | 11.63039157 | -4.736117619 | 6.21E-06 | 0.000409764 | 3.641783827 |
|  | CKAP4 | 0.186536176 | 7.399521713 | 3.830750362 | 0.000207586 | 0.005557205 | 0.374989942 |
|  | DPEP2 | 0.175508156 | 5.494866765 | 3.37574186 | 0.001001424 | 0.016888254 | -1.066564558 |
