# Supplementary material for: Molecular mechanisms of lipid metabolism abnormalities driving sepsis and atrial fibrillation: A Systematic study based on bioinformatics and machine learning
Source: PLoS One. 2025 Dec 8;20(12):e0338511. doi: 10.1371/journal.pone.0338511 (PMC12685191; doi:10.1371/journal.pone.0338511)
Supplement: Attachment — Submitted filename: Response to Reviewer1.docx [file pone.0338511.s004.docx]

Response to Reviewers' Comments – Manuscript ID [PONE-D-25-28089R1]

Dear Reviewer1,

Thank you for your valuable feedback and constructive suggestions on our manuscript titled "Molecular Mechanisms of Lipid Metabolism Abnormalities Driving Sepsis and Atrial Fibrillation: A Systematic Study Based on Bioinformatics and Machine Learning". We have carefully revised the manuscript in accordance with your recommendations. Below, we provide a detailed point-by-point response to your comments.

1. Clarify dataset flow and harmonization: add a one-page flow diagram and briefly describe batch correction or use of study covariates. A simple schematic that lists platform, tissue, n, discovery vs validation, and QC would help a lot.

Response: To address this issue, we have supplemented Supplementary Figure 1, which intuitively illustrates the complete analytical workflow, ranging from dataset acquisition, QC, and batch correction to model training and validation. Concurrently, we have updated Table 1 (Dataset Information) to explicitly add "Tissue Source" (e.g., whole blood for sepsis datasets, right atrial appendage/left atrial appendage for AF datasets) and "Cohort Role" (discovery/training set, validation set, single-cell analysis set) for each GSE series. For scRNA-seq data (GSE167363), we have refined the QC criteria in the "Single-Cell Transcriptome Analysis" section of the Methods. Specifically, we have clarified the thresholds for cell retention (e.g., nFeature_RNA > 300, mitochondrial gene proportion < 20%) and the rules for outlier exclusion. Regarding batch correction, additional details have been supplemented in the "Batch-Corrected Integration of AF Datasets" section: the removeBatchEffect function from the R package limma was employed, with "dataset origin + detection platform" set as covariates to eliminate systematic biases. The effectiveness of batch correction was verified using PCA and box plots of gene expression distribution.

2. Standardize or justify DEG thresholds: align to a common |logFC| (for example, ≥0.5) or add a short rationale plus a quick sensitivity check to show stability of key results.

Response: The threshold setting for DEGs was based on the unique transcriptomic characteristics of the two diseases, which has been further elaborated in the "Differential Analysis of Gene Expression Data" section: Sepsis: As a disease driven by acute inflammatory storms, it exhibits drastic fluctuations in gene expression. A threshold of |logFC| > 1 was adopted to filter out low-amplitude background noise and prioritize the screening of high-confidence genes associated with inflammatory cascades. AF: As a disease characterized by chronic, low-magnitude electrophysiological and structural remodeling, key molecular events (e.g., genes regulating atrial collagen deposition or calcium handling) often present with subtle changes in expression. Therefore, a threshold of |logFC| > 0 was used to avoid missing such biologically meaningful genes. Indicators including the logFC values, average expression levels, and statistical significance (adjusted P-values) of the three core hub genes (CD81, CKAP4, and DPEP2) have been analyzed and presented in Table 3.

3. Specify and lock the validation pipeline: say where feature selection sits relative to cross-validation, how hyperparameters were tuned, and confirm that external sets were untouched until final testing. Add PR-AUC and a simple calibration metric (Brier) in the supplement. A brief leave-one-study-out check would be a nice bonus.

Response: Feature selection was strictly embedded in a nested 10-fold cross-validation (CV) framework: the inner loop implemented feature ranking and hyperparameter tuning (gamma: 2^(-12:0), cost: 2^(-6:6)) for support vector machine-recursive feature elimination (SVM-RFE) using training subsets, while LASSO regression determined the optimal regularization parameter λ (λ_min) via 10-fold CV to minimize cross-validation deviance, and the outer loop used independent test subsets to evaluate generalization error, ensuring no data leakage between training and testing phases. External validation sets (GSE65682 for sepsis, GSE41177 for atrial fibrillation) were completely isolated during model training, feature selection, and parameter optimization, and only used for final diagnostic performance evaluation (e.g., ROC curve analysis) to avoid overfitting. The original manuscript used ROC-AUC (area under the receiver operating characteristic curve) to assess diagnostic efficacy (sepsis: 0.957, atrial fibrillation: 1.000), a widely recognized metric with clinical interpretability for binary classification tasks in diagnostic research. Due to the initial focus on clinically relevant metrics, PR-AUC (area under the precision-recall curve) and Brier score (a calibration metric quantifying the discrepancy between predicted probabilities and actual outcomes) have not yet been included in the supplementary materials, but will be added in future studies to comprehensively evaluate model performance; leave-one-study-out cross-validation (LOSO-CV) was not conducted in the current work, and will be considered in subsequent research to further validate the model’s generalizability.

4. Validate the lipid gene universe: repeat enrichment and selection with curated Reactome or GO or KEGG lipid sets and report whether top pathways and hubs remain consistent. A short methods note plus a small supplemental table is enough.

Response: The key genes in this study have not yet been included as "direct lipid metabolism-related genes" in curated databases such as Reactome; however, these genes are indirectly involved in lipid metabolism by regulating upstream and downstream lipid metabolism signals (e.g., inflammation-mediated lipolysis, lipid transport in immune cells). In contrast, the GeneCards database, which incorporates genes with such indirect regulatory roles, offers broader coverage.

5. Detail immune deconvolution and scRNA-seq QC: name the signature or tool, note how multiple testing across cell types was handled, and list basic scRNA thresholds (UMIs or genes, mitochondrial percent, doublet handling). One concise paragraph in Methods will do.

Response: For immune deconvolution, a modified CIBERSORT algorithm was used with the LM22 signature matrix (containing specific gene markers for 22 human immune cell subtypes) as reference; data preprocessing included quantile normalization, batch effect correction, and z-score normalization of overlapping genes, while the Benjamini-Hochberg method was applied to adjust P-values for multiple tests across cell types, and credible samples were filtered with false discovery rate (FDR) < 0.05 and root mean square error (RMSE) < 0.1. For single-cell RNA sequencing (scRNA-seq) quality control of GSE167363, cells were retained based on the following criteria: nFeature_RNA > 300 (to exclude low-quality cells with sparse gene detection), mitochondrial gene proportion < 20% (to exclude cells with severe mitochondrial damage), and nCount_RNA < 6000 (for preliminary doublet exclusion); doublets were further identified by analyzing the correlation between nCount_RNA and nFeature_RNA, with cells showing abnormally high nCount_RNA relative to nFeature_RNA classified as potential doublets and removed.

6. Reframe drug-enrichment as exploratory: present DSigDB or Enrichr hits as hypotheses and, if possible, cross-check with an orthogonal resource such as L1000 or CMap. Avoid therapeutic language.

Response: We have fully revised the "Exploratory Compound Enrichment Analysis" section to emphasize the exploratory nature of the results and avoid overinterpretation as therapeutic candidates: the title was changed from "Potential Drug Prediction" to "Exploratory Compound Enrichment Analysis" to reflect its hypothesis-generating purpose, and descriptive language was adjusted—replacing "candidate drugs with therapeutic potential" with "exploratory compound-gene interaction hypotheses"—while clarifying that the analysis was based on gene-compound association data from the DSigDB database (retrieved via the Enrichr platform), aiming to identify potential molecular interaction directions rather than confirm therapeutic efficacy.

7. Qualify performance claims: keep the strong AUCs but add a brief caveat about cohort size and platform or tissue heterogeneity. Include study-wise curves in the supplement so readers can see consistency.

Response: A brief caveat regarding cohort size and platform or tissue heterogeneity has been added to the Discussion section. In subsequent work, we will generate study-level ROC curves based on existing data to intuitively demonstrate the consistency of results across different datasets, thereby enhancing the credibility of the conclusions.

8. Qualify performance claims: keep the strong AUCs but add a brief caveat about cohort size and platform or tissue heterogeneity. Include study-wise curves in the supplement so readers can see consistency.

Response: At present, the aforementioned materials have not yet been uploaded to a public repository. For immediate access, interested parties may contact the corresponding author directly, and we will promptly provide the complete materials to support the reproducibility of this study.

Once again, we sincerely thank you for your meticulous review and insightful suggestions. These revisions have significantly improved the rigor, clarity, and transparency of our study. We believe the revised manuscript will make a more valuable contribution to understanding the shared mechanisms of lipid metabolism abnormalities in sepsis and AF.

Sincerely,

The Authors
